# Minimizing Extrinsic Effects in High-Pressure Raman of Monolayer WSe_2_ through Substrate and Pressure-Transmitting Medium Control

**DOI:** 10.1021/acsomega.5c12301

**Published:** 2026-02-11

**Authors:** Jose Hugo Aguiar Sousa, Ramon S. Ferreira, Alexandre Cavalheiro Dias, Ian Rodrigues do Amaral, Alfonso San-Miguel, Rafael S. Alencar, Antonio G. Souza Filho

**Affiliations:** † Departamento de Física, 28121 Universidade Federal do Ceará, Fortaleza, CE 60455-900, Brazil; ‡ Departamento de Física, 67823 Universidade Federal do Piauí, Teresina, PI 64049-550, Brazil; § 564113 University of Brasília, Institute of Physics and International Center of Physics, Brasília 70919-970, Brazil; ∥ Univ Lyon, 27098 Université Claude Bernard Lyon 1, CNRS, Institut Lumière Matière, F-69622 LYON, France

## Abstract

We present a comprehensive study of the Raman spectra of monolayer WSe_2_ under high pressure up to 40 GPa, focusing on the influence of both the substrate and the pressure-transmitting medium (PTM). By using diamond as the substrate, which minimizes strain transfer compared to conventional Si/SiO_2_, we isolate the intrinsic vibrational response of monolayer WSe_2_ from substrate-induced effects. Our results show that the enhancement of LA-related second-order modes occurs only at pressures >15 GPa, much higher than in Si/SiO_2_-supported samples, reflecting the weaker strain coupling to diamond. However, once the PTM loses hydrostaticity, non-uniform strain rapidly intensifies these modes. The role of strain-induced disorder is further evidenced by the appearance of wrinkles after decompression, which lead to local symmetry breaking and activate the normally forbidden B_2*g*_ mode. Density functional theory (DFT) calculations indicate that the K-Λ valley crossover in the conduction band occurs at higher pressures than previously reported, but still much lower than those required to tune the second-order modes, thus suggesting that their intensification is predominantly driven by strain-induced disorder rather than electronic transitions. These findings provide practical guidelines for minimizing substrate- and PTM-related artifacts, thereby enabling more accurate and reproducible high-pressure Raman characterization of two-dimensional materials.

## Graphical abstract

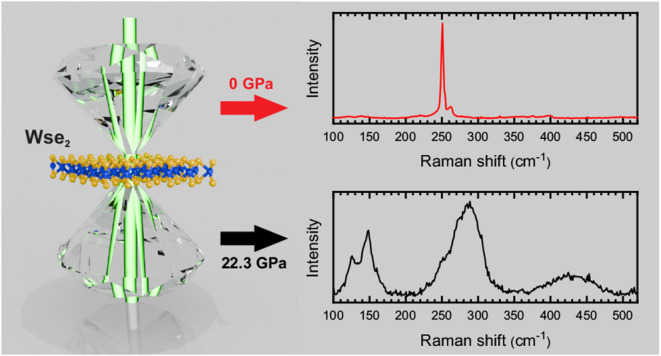

## Introduction

Two-dimensional (2D) transition metal dichalcogenides (TMDs), described by the formula MX_2_ (M = W, Mo; X = S, Se), have emerged as model systems for investigating quantum confinement, excitonic effects, and valley physics at the atomic scale.^1–4^ In particular, monolayer tungsten diselenide (1L-WSe_2_) has attracted significant attention as a direct-gap semiconductor with strong light-matter interaction, high carrier mobility, and pronounced spin–orbit coupling, making it a promising candidate for optoelectronics and valleytronic applications.^5–10^ Since the physical properties of 2D materials are highly sensitive to external thermodynamic perturbations,^11–17^ hydrostatic pressure has become a valuable tool to continuously tune their structural, optical, and electronic properties without introducing chemical modification.^18–23^

Raman spectroscopy is a well-established technique for probing lattice dynamics, strain, and electronic structure in TMDs under high-pressure conditions.^19–21,23,24^ Previous studies have shown that compression induces systematic frequency shifts and intensity variations not only in first-order phonons^19,24^ but also in second-order Raman features.^20,25^ The pronounced pressure-induced enhancement of these higher-order modes has been attributed either to double-resonance Raman (DRR) processes or to disorder-assisted scattering,^20,25^ yet the dominant mechanism remains under debate.

A critical factor complicating the interpretation of high-pressure studies in 2D systems is the experimental environment. Diamond anvil cell experiments often assume hydrostatic conditions, but this assumption becomes less reliable for monolayers supported on substrates. In such cases, pressure-induced substrate deformation can transfer biaxial strain to the 2D layer, modifying phonon frequencies and scattering intensities.^23,26,27^ Moreover, loss of hydrostaticity in the PTM can introduce anisotropic stress components that further alter the Raman response to a great extent.^27–30^ These extrinsic effects may mask intrinsic lattice behavior or even generate misleading responses, underscoring the need to separate substrate- and PTM-induced artifacts from true vibrational and electronic modifications.^31^ Addressing these experimental factors is therefore essential to establish reliable protocols for probing structure—property relationships in layered semiconductors under extreme conditions.

In this work, we investigate the Raman response of monolayer WSe_2_ under high pressure, with particular emphasis on the roles of the substrate and the PTM. By employing diamond as a substrate, which minimizes strain transfer compared to conventional SiO_2_, and by carefully monitoring the hydrostaticity of the 4:1 methanol: ethanol mixture PTM, we clarify how environmental factors govern the evolution of first- and second-order Raman modes. This combined approach provides new insights into the interplay between substrate coupling, hydrostatic conditions, and vibrational dynamics, thereby contributing to more accurate and reproducible interpretation of high-pressure Raman data in atomically thin semiconductors.

## Results and Discussion

Figure 1a shows an optical image of the 1L-WSe_2_ (outlined by dashed white lines) placed on the diamond culet of the diamond anvil cell (DAC) high-pressure apparatus. The characteristic Raman spectrum of the system at ambient pressure and room temperature is presented in Figure 1b. Each vibrational mode was fitted with a Lorentzian function. The in-plane E_2*g*_(Γ) and out-of-plane A_1*g*_(Γ) modes are nearly degenerate, both appearing at frequencies around 250 cm^–1^. The absence of the B_2*g*_(Γ) mode at 308 cm^–1^ confirms the monolayer nature of the sample.^32^

**Figure 1:**
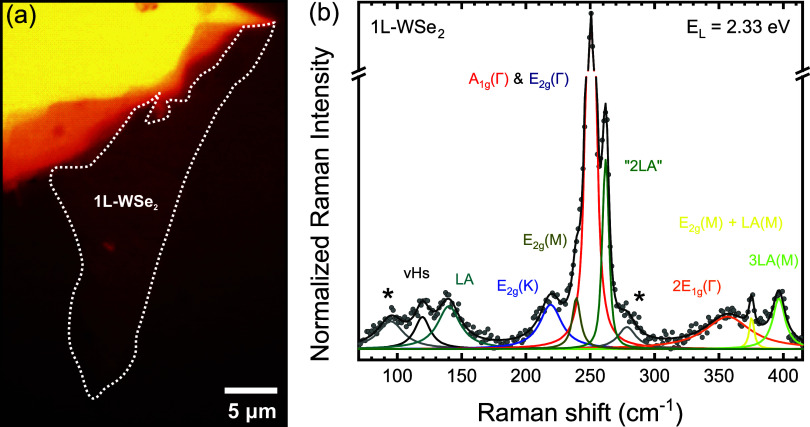
(a) Optical image of the WSe_2_ flake deposited on the diamond plateau. The white dashed line marks the monolayer region. (b) Raman spectrum of 1L-WSe_2_ acquired under ambient conditions (room temperature and atmospheric pressure) with a 2.33 eV laser excitation. Star symbols denote auxiliary peaks introduced solely to improve the fitting accuracy, without correspondence to intrinsic Raman modes of WSe_2_.

Beyond the first-order modes at the Brillouin zone center, several second-order modes and overtones are also present, labeled following refs 20,33,34 . These include the vHs feature at ∼120 cm^–1^, and the LA­(M) and LA­(K) peaks, which are here collectively referred to as the LA band centered at ∼140 cm^–1^; E_2*g*_(K) and E_2*g*_(M), centered at 225 and 242 cm^–1^, respectively; and 2E_1*g*_(Γ), E_2*g*_(M) + LA­(M), and 3LA­(M), centered at 360, 371, and 394 cm^–1^, respectively.

The band around 260 cm^–1^, often referred to as “2LA” in the literature, actually comprises at least three components: 2vHs, 2LA­(M), and 2LA­(K), centered at approximately 258, 260, and 263 cm^–1^, respectively. The LA­(M) and LA­(K) modes originate from the longitudinal acoustic (LA) phonon branch at the M or K points of the Brillouin zone, typically activated by structural disorder in the lattice.^25^ The 2vHs component is associated with two-phonon scattering resulting from a van Hove singularity (vHs) at the saddle point in the phonon density of states, located between the K and M points of the longitudinal acoustic phonon dispersion branch.^20^ Although the “2LA” band consists of three components, we used a Voigt function to fit the “2LA” band across the entire pressure range for a more reliable analysis of this feature.

Figure 2a presents the Raman spectrum of 1L-WSe_2_ at several pressures (the complete pressure cycle is shown in Figure S1 of the Supporting Information (SI)). Both first-order and second-order modes broaden and shift to higher frequencies as the pressure increases. In particular, the intensity of the LA and vHs modes increases significantly at pressures higher than 15 GPa. To better visualize weak-intensity Raman features while preserving the uniform intensity scale used in Figure 2a, the spectra plotted with an expanded intensity scale are shown in Figure S2 of the SI.

**Figure 2:**
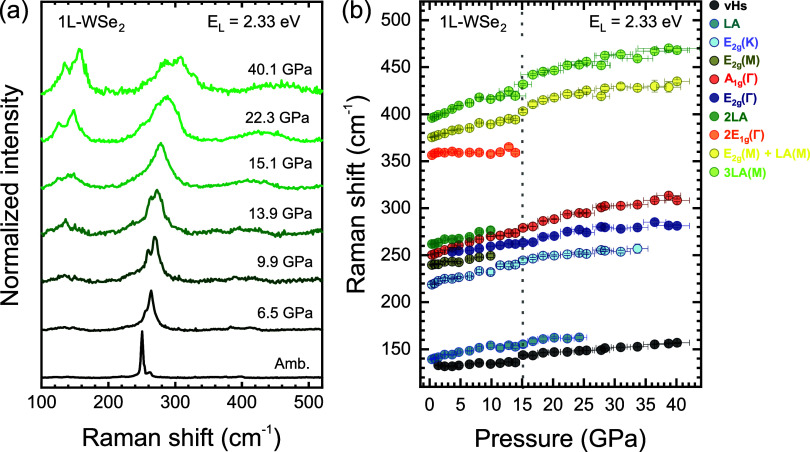
(a) Raman spectra of 1L-WSe_2_ recorded at different pressure conditions. (b) Pressure dependence of the vibrational modes, as labeled in Figure 1b.

Figure 2b displays the Raman frequencies of the vibrational modes as a function of pressure. The peaks increase monotonically with pressure up to approximately 15 GPa, as indicated by the dashed line. At this pressure, a discontinuity appears in the pressure coefficients of all modes. Analysis of the ruby fluorescence band (see Figure S3 in the SI) reveals that the PTM undergoes a glass transition at this point, substantially modifying the hydrostatic conditions of the experiment. This transition introduces strain components into the 1L-WSe_2_ lattice, thereby affecting the pressure dependence (∂ω/∂*P*) of each phonon mode and contributing to the apparent disappearance of the LA and 2E_1*g*_(Γ) modes. This apparent disappearance arises from peak broadening, reduced signal-to-noise ratio, and overlap with neighboring second-order features, which prevent reliable peak tracking in this pressure range.

The increase in the intensity of the second-order modes, particularly the LA band and its overtone, with increasing pressure, has been studied in refs 25 and 20. In addition to the pressure regime, in which the intensification of the second-order modes occurs, the authors also diverge in the interpretation of the results. Pimenta Martins et al.^20^ attributed the enhancement of the second-order modes under pressure to a double resonance Raman effect. As pressure increases, it tunes the resonance of exciton B (the higher-energy exciton arising from spin—orbit splitting) and the indirect transition between the K and Q points (denoted here as Λ) of the first Brillouin zone (by opening the direct gap at the K point and decreasing Δ*E*_KΛ_).

In contrast, Gong et al.^25^ suggest that the increase in intensity of the second-order modes is due to structural disorder, as the intensity of the LA mode is closely related to the degree of disorder in the crystalline lattice of the sample.^20,35–37^ Typically, lattice defects and unintentional doping are the primary causes of the appearance of an LA peak under ambient pressure conditions.^25^ As pressure increases, the doping level was assumed to remain constant in that work. Therefore, the increase in the LA peak is attributed to pressure-induced lattice distortion rather than doping.^25^

In high-pressure experiments, it is generally assumed that a hydrostatic or quasi-hydrostatic pressure is transmitted from the PTM to the sample. This concept is well-suited for bulk (3D) layered materials, as the PTM surrounds the entire sample. However, in 2D systems, they are often studied under pressure while being deposited on substrates. In this scenario, the assumption of hydrostatic pressure is invalid, since the deformation of the substrate due to pressure induces biaxial in-plane strain to the 2D material in regions of perfect adhesion, which depends on the relative bulk modulus of the sample and the substrate.^23,26,27^ When the substrate’s in-plane bulk modulus is significantly lower than that of the sample, strain transfer is important. For instance, it has been shown that MoS_2_ deposited on SiO_2_ experiences substantial strain transfer through substrate deformation, which is critical in the structural evolution of the TMD.^23^

In the studies by Gong et al.^25^ and Martins et al.,^20^ SiO_2_ was used as the substrate for depositing monolayer WSe_2_. This setup introduces a strain component from substrate deformation. Consequently, the intensification of the second-order modes occurs at lower pressures compared to our results. In ref 20, the LA and 2LA modes reach approximately half the intensity of the A_1*g*_ mode at 3.5 GPa. In ref 25, the LA mode reaches half the intensity of the A_1*g*_ mode at 12.2 GPa. However, in our experiment, the LA mode achieves half the intensity of the A_1*g*_ mode only at around 22.3 GPa, as shown in Figure S4a of the SI. This pressure difference is due to the use of diamond as a substrate for the 1L-WSe_2_ sample. With a linear bulk modulus of approximately 1220 GPa,^38^ this setup results in an induced strain component nearly 16 times smaller than the strain experienced by 1L-WSe_2_ on a SiO_2_ substrate, with ∼80 GPa.^39^ On the other hand, when the PTM becomes nonhydrostatic, the effect of strain (now caused by the PTM) rapidly increases the intensity of the LA mode.

Additional evidence supporting the interpretation that structural defects contribute to the intensification of the LA mode is the formation of wrinkles in the 1L-WSe_2_ sample after the pressure cycle, as shown in Figure 3. The origin of these wrinkles lies in the difference in compressibility between the sample and the substrate. While the diamond linear bulk modulus is very large (∼1220 GPa), the WSe_2_ linear bulk modulus is approximately 20 times smaller, i.e., ∼63 GPa.^40^ The consequence of this difference is not evident during the compression phase, as the sample compresses at a higher rate, but during decompression. With a higher expansion rate, and despite the low general adhesion between sample and substrate, widespread wrinkles appear in scattered adhesion points, resulting in the observed structural defects. Pressure-induced wrinkle formation has also been reported in graphene,^28^ and this interesting (reversed) phenomenon is also observed here.

**Figure 3:**
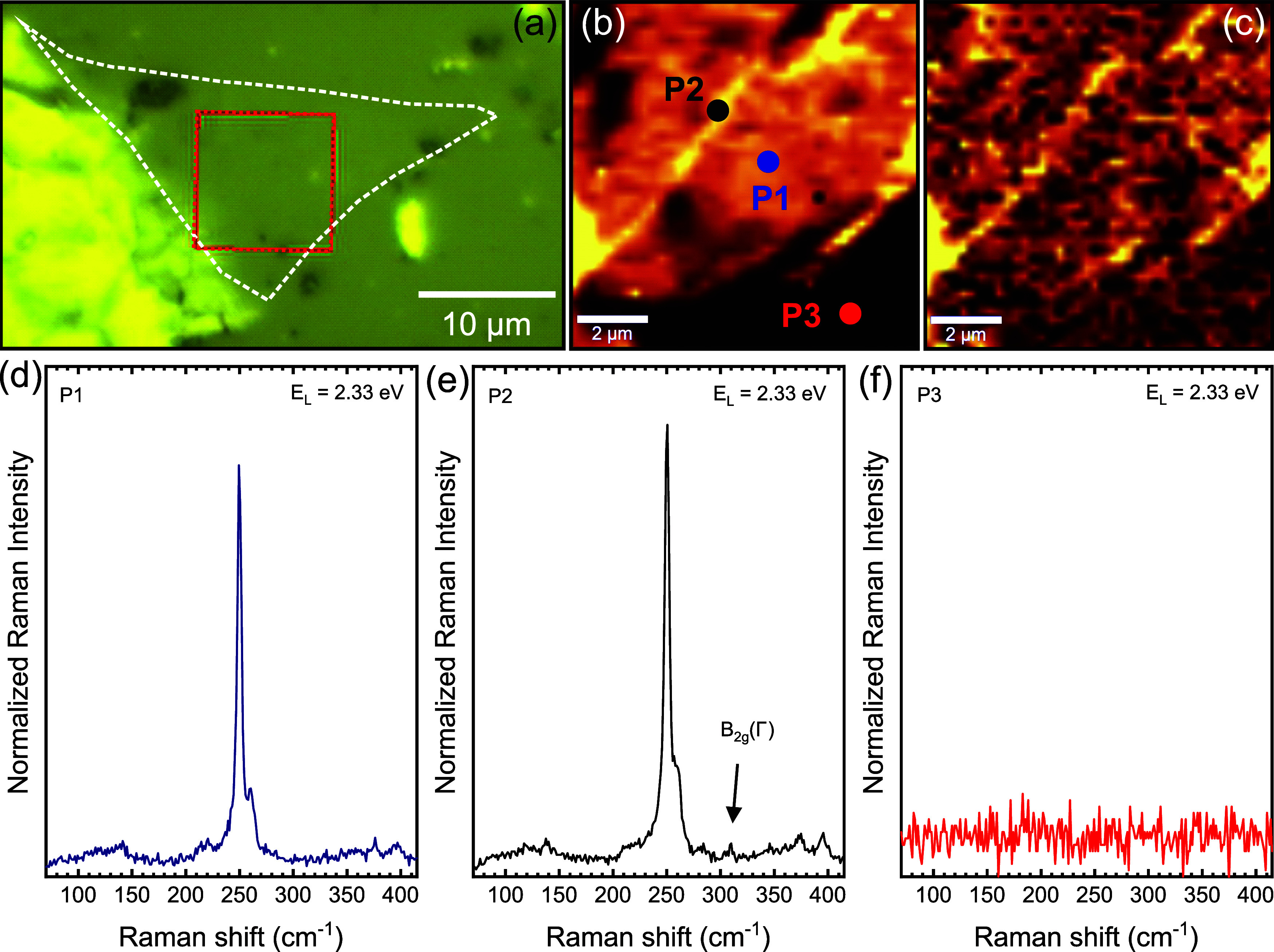
(a) Optical image of the WSe_2_ flake on the diamond culet at ambient conditions, after the complete pressure cycle up to 40 GPa. The white dashed line marks the monolayer region, and the red square indicates the area mapped in (b) and (c). (b) Raman map of the A_1*g*_ mode. (c) Raman map of the B_2*g*_ mode. (d–f) Raman spectra recorded at positions P1, P2, and P3 marked in panel (b), respectively.

Figure 3b presents a Raman map of the A_1*g*_ mode from the region marked by the red square in panel (a). Brighter features in this map are consistent with wrinkle positions, suggesting that local strain associated with the wrinkles enhances the A_1*g*_ intensity. Moreover, these wrinkles can activate the B_2*g*_ mode, which is normally forbidden in monolayer WSe_2_ but becomes Raman-active due to symmetry breaking. This effect is supported by Figure 3c and e, which correspond to the Raman map and a representative Raman spectrum of the B_2*g*_ mode, respectively. The B_2*g*_ peak is observed at regions attributed to wrinkles, while it is absent in spectra collected away from the wrinkles (Figure 3d and f). This difference is further illustrated in Figure S4b of the SI.

Although the wrinkle analysis presented here is primarily qualitative, a rough estimate of the local strain can be obtained from the Raman frequency shifts between wrinkle and flat regions. In monolayer WSe_2_, the near degeneracy of the E_2*g*_ and A_1*g*_ modes limits direct strain calculation from Raman spectra, since the A_1*g*_ mode exhibits a weaker strain sensitivity than the E_2*g*_ mode, and polarization-resolved Raman measurements are required to fully separate their individual contributions. In the absence of polarization-resolved measurements, we follow the approach reported by Michail et al.,^41^ who determined an effective biaxial strain coefficient of approximately 1.45 cm^–1^/% for the degenerate A_1*g*_/E_2*g*_ mode in monolayer WSe_2_. By comparing the Raman spectra acquired at wrinkle sites and flat areas, we observe a frequency difference of about 0.28 cm^–1^ for the A_1*g*_/E_2*g*_ peak, which corresponds to an effective local compressive strain of approximately 0.19%. Given the localized and nonuniform nature of the strain at wrinkle regions, this value should be regarded as an order-of-magnitude estimate. It is important to note that this effective average strain is not sufficient, by itself, to break the lattice symmetry. The activation of the B_2*g*_ mode is instead attributed to strain gradients and out-of-plane curvature associated with wrinkle formation, which locally reduce the crystal symmetry and relax the Raman selection rules.

Additionally, because the double-resonance scattering process requires resonance conditions (namely involving both direct transitions at the K point and indirect transitions between the K and Λ points), one would expect a crossover from direct to indirect band gap feature above 15 GPa. In this sense, we carried out DFT calculations to examine the pressure dependence of the electronic band structure of monolayer WSe_2_ (Figure 4). Our analysis focused on the B exciton (X_*B*_), which lies closest to the laser excitation energy used in this study (2.33 eV), and we restricted the discussion to spin-conserving transitions, following ref 20. The calculations indicate that the indirect transition involving the K and Λ valleys (highlighted by arrows in Figure 4) of the conduction band becomes energetically favorable at 6.0 GPa, higher than was observed in previous reports.^12,20,25^

**Figure 4:**
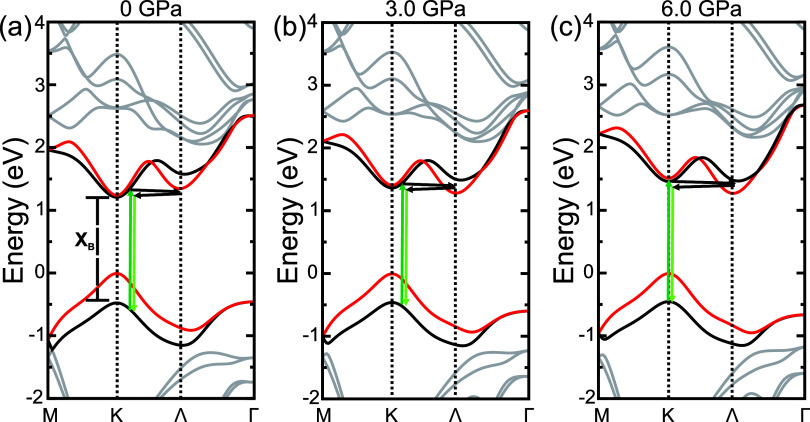
(a–c) Calculated pressure-induced K−Λ valley crossover in monolayer WSe_2_. Spin polarization is indicated (red: ↓, black: ↑). Arrows illustrate the scattering process involving optical excitation at K, intervalley K → Λ scattering, and B exciton recombination.

Although the calculations are performed at 0 K, they reliably capture the relative pressure evolution of the electronic band structure. In particular, the calculated K−Λ crossover at 6.0 GPa occurs at a pressure substantially lower than the onset of the pronounced enhancement of the LA-related double-resonance Raman features, which is experimentally observed only above ∼15 GPa. This large pressure mismatch cannot be accounted for by thermal effects between 0 K and room temperature and therefore rules out an electronic-resonance-driven mechanism. Consequently, the intensity increase of the second-order modes is attributed to disorder effects associated with strain, which are further amplified by the loss of hydrostaticity in the pressure-transmitting medium. While ref 20 interpreted the enhancement of double-resonance modes as arising from the combined effects of K-Λ valley crossing and the blueshift of the B exciton toward resonance with the excitation energy, our results point to a regime in which strain-induced disorder plays the dominant role.

## Conclusion

In summary, the high-pressure Raman response of monolayer WSe_2_ was investigated with emphasis on substrate coupling and PTM conditions. The use of diamond as substrate reduced the transfer of strain in relation to SiO_2_, thus allowing the intrinsic pressure evolution of the Raman modes to be accessed. The enhancement of LA-related second-order modes was observed only above 15 GPa, a much higher pressure than in SiO_2_-supported samples, thus reflecting weaker strain coupling to diamond. Once the PTM lost hydrostaticity, non-uniform strain components strongly intensified these modes. After decompression, wrinkle formation activated the normally forbidden B_2*g*_ mode, confirming the role of strain-induced disorder. DFT calculations indicated that the K−Λ valley crossover occurs at higher pressures than previously reported but still far below those required to explain the observed Raman intensification, showing that disorder rather than electronic transitions dominates this effect. Overall, these results establish substrate and PTM conditions as decisive factors and provide practical guidelines for the reliable interpretation of high-pressure Raman studies in two-dimensional semiconductors.

## Materials and Methods

### Sample Preparation

A monolayer WSe_2_ flake was prepared by mechanical exfoliation of bulk crystals onto a polydimethylsiloxane (PDMS) substrate and subsequently transferred in a controlled manner onto the diamond culet of the DAC. The PDMS stamp was used exclusively during the exfoliation and transfer process and was completely removed after the monolayer was deposited on the diamond culet. During high-pressure measurements, the monolayer is directly supported by the diamond surface, which therefore acts as the mechanical substrate. The use of diamond as a supporting substrate significantly reduces strain transfer compared to conventional SiO_2_, enabling a more reliable characterization of the intrinsic pressure response of the monolayer. The monolayer regions were first identified by their characteristic optical contrast and further confirmed by Raman spectroscopy at ambient pressure, where the absence of the B_2*g*_ mode (normally present in multilayer WSe_2_) served as a fingerprint of the single-layer nature.

### High-Pressure Setup

High-pressure experiments were performed using a membrane-driven DAC with a culet size of ∼350 μm. A cylindrical pressure chamber of ∼150 μm in diameter was drilled into a pre-indented stainless-steel gasket, which was subsequently loaded with the 1L-WSe_2_ sample, ruby spheres for pressure calibration, and the PTM. A 4:1 methanol–ethanol mixture was used as the PTM, since it is known to remain liquid and hydrostatic above 10 GPa in a metastable liquid state.^42^ Pressure values were determined from the shift of the ruby *R*_1_ luminescence line.

### Raman Measurements

Raman spectra were acquired in backscattering geometry using a confocal micro-Raman WITec Alpha300 spectrometer with a laser excitation energy of 2.33 eV. The laser power was maintained at 2.5 mW at the output of a 20× magnification objective lens (NA = 0.35) to minimize local heating, prevent photoinduced damage, and improve the signal-to-noise ratio. The scattered light was dispersed using a grating with 1800 grooves/mm, providing a spectral resolution of ±1.0 cm^–1^.

### First-Principles Calculations

First-principles calculations were performed within the framework of DFT as implemented in the Vienna *Ab initio* Simulation Package (VASP).^43,44^ The projector-augmented wave (PAW) method^45^ was employed to describe the electron–ion interactions. The exchange–correlation potential was treated within the generalized gradient approximation (GGA) using the Perdew–Burke–Ernzerhof (PBE) functional.^46^

Relativistic effects were incorporated by including spin–orbit coupling (SOC) in all calculations. A plane-wave kinetic energy cutoff of 400 eV was employed. The electronic self-consistency loop was converged to a tolerance of 10^–6^ eV. For the geometry optimization, a conjugate-gradient algorithm was used to relax both the atomic positions and the unit cell shape and volume until the Hellmann–Feynman forces on each atom were less than 0.01 eV/Å. The Brillouin zone (BZ) was sampled using a 32 × 32 × 1 Γ-centered Monkhorst–Pack *k*-point grid,^47^ and a Gaussian smearing of 0.01 eV was applied. To avoid spurious interactions between adjacent layers, a vacuum spacing of approximately 20 Å was introduced along the nonperiodic direction.

To generate the structures under hydrostatic pressure, the bulk lattice of WSe_2_ was subjected to an equivalent external stress. The electronically and structurally optimized strained bulk structure was then cleaved to obtain a single monolayer of WSe_2_. This procedure preserves the in-plane lattice parameters corresponding to the applied pressure, thereby effectively simulating hydrostatic conditions in the monolayer. The process was repeated for each pressure value investigated in this work. A similar strategy to construct hydrostatically strained structures has also been employed in previous studies.^48–50^

## Supplementary Material



## Notes

Additional Raman spectra under pressure, ruby fluorescence analysis for hydrostaticity evaluation, pressure dependence of the LA/A_1*g*_&E_2*g*_ intensity ratio, and stacked Raman spectra from wrinkle and flat regions (PDF)
