# Supplementary material for: Minimizing Extrinsic Effects in High-Pressure Raman of Monolayer WSe2 through Substrate and Pressure-Transmitting Medium Control
Source: ACS Omega. 2026 Feb 11;11(7):12472–8. doi: 10.1021/acsomega.5c12301 (PMC12947151; doi:10.1021/acsomega.5c12301)
Supplement: Supplementary file 1 [file ao5c12301_si_001.pdf]

Support information for "Minimizing Extrinsic Effects in High-Pressure Raman of Monolayer WSe_2 through Substrate and Pressure-Transmitting Medium Control"

Jose Hugo Aguiar Sousa,[†] Ramon S. Ferreira,[‡] Alexandre Cavalheiro Dias,[¶] Ian Rodrigues do Amaral,[†] Alfonso San-Miguel,[§] Rafael S. Alencar,[*,†] and Antonio G. Souza Filho[*,†]

†*Departamento de Física, Universidade Federal do Ceará, Fortaleza, CE, 60455-900 Brazil*

‡*Departamento de Física, Universidade Federal do Piauí,Teresina, PI, 64049-550 Brazil*

¶*University of Brasília, Institute of Physics and International Center of Physics, Brasília, Brazil*

§*Univ Lyon, Université Claude Bernard Lyon 1, CNRS, Institut Lumière Matière, F-69622 LYON, France*

E-mail: rafael_alencar@fisica.ufc.br; agsf@fisica.ufc.br

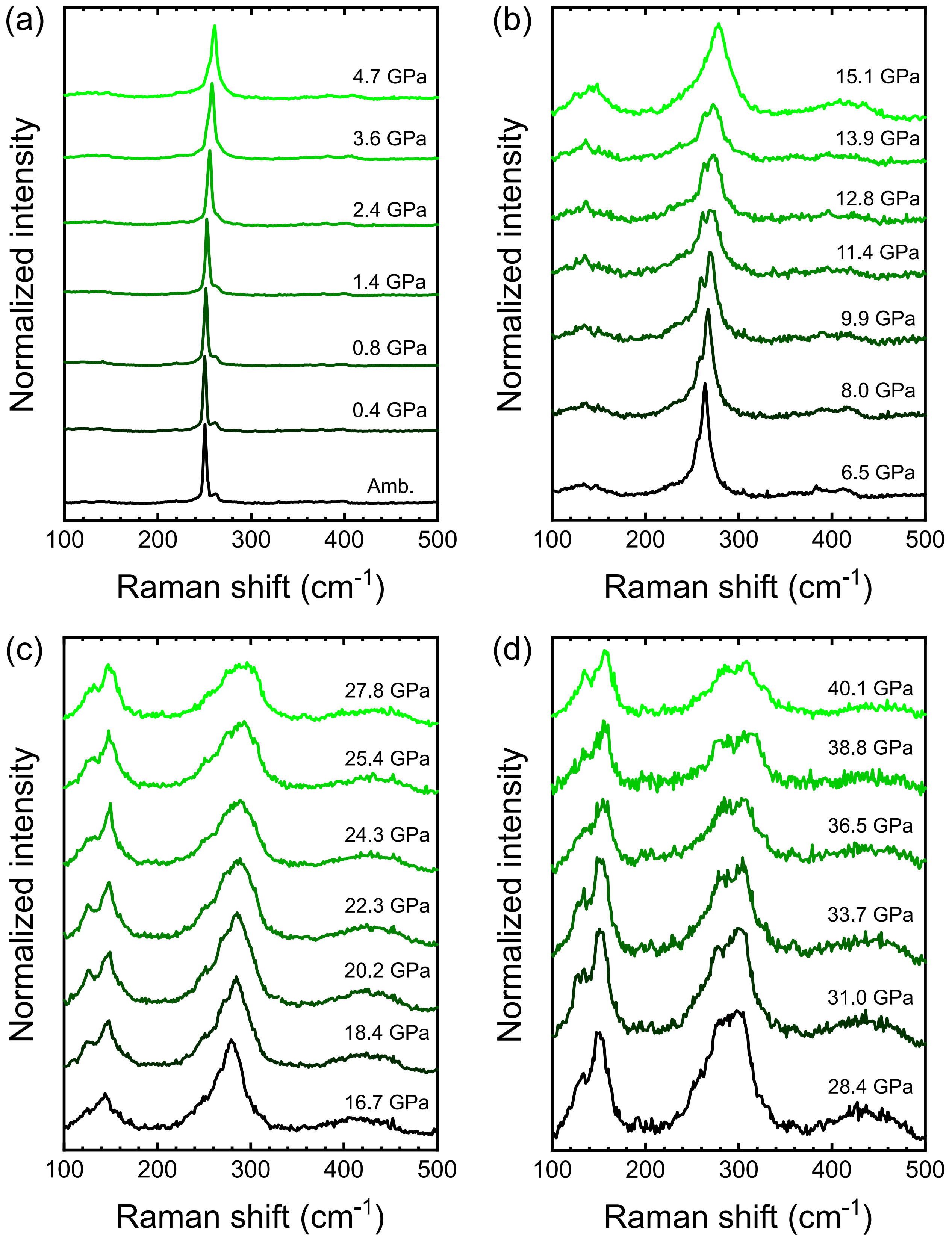

Figure S 1: (a-d) Raman spectra of 1L-WSe_2 obtained at different pressure values.

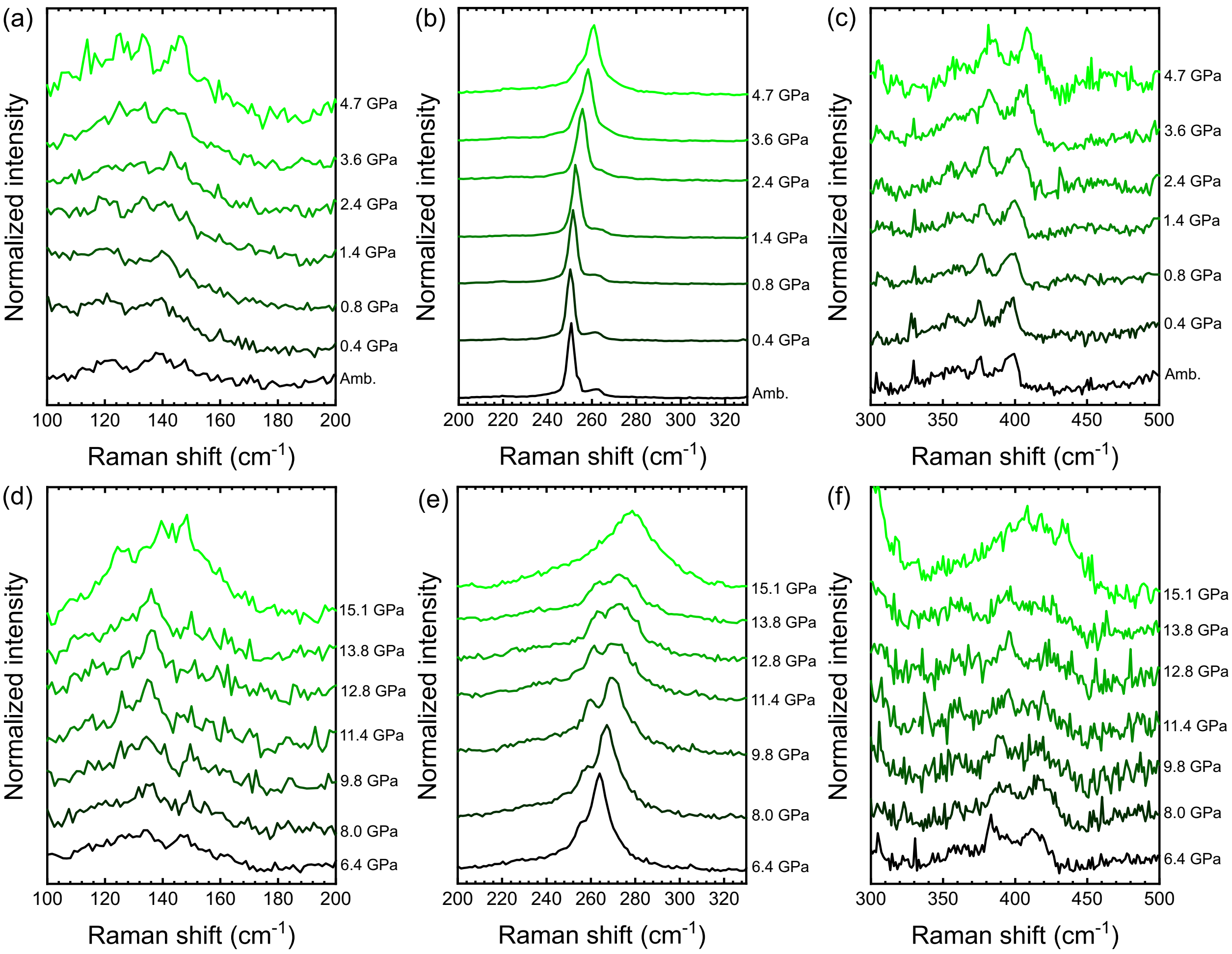

Figure S 2: Raman spectra of 1L-WSe_2 obtained at different pressure values, plotted with an expanded intensity scale to enhance the visibility of weak-intensity features. The spectra are divided into the 100–200 cm^{-1} (a,d), 200–330 cm^{-1} (b,e), and 300–500 cm^{-1} (c,f) spectral regions and vertically stacked up to 15.1 GPa.

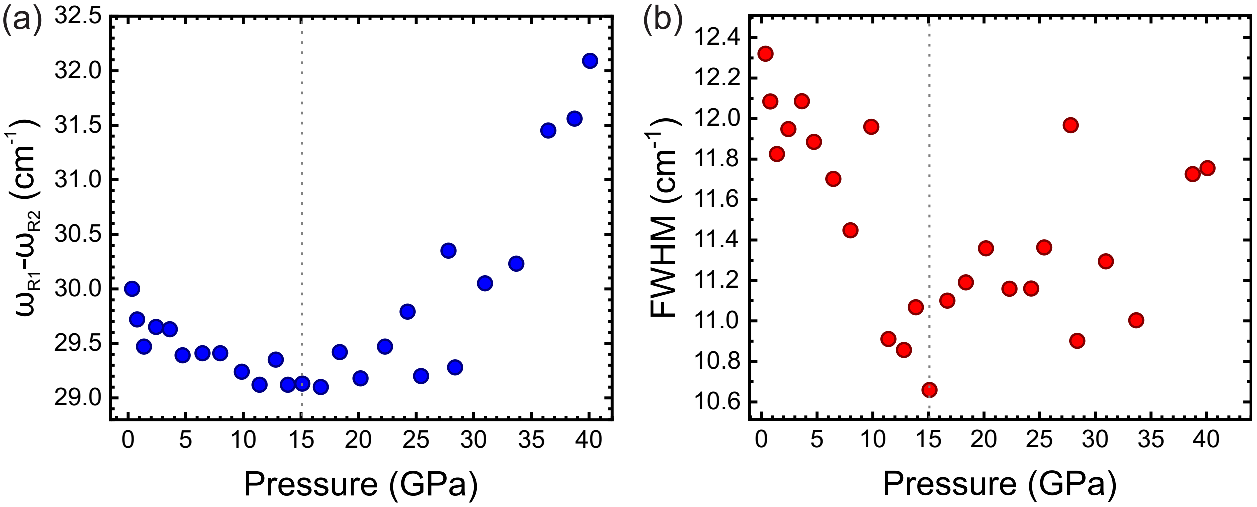

Figure S 3: Pressure dependence of the (a) frequency difference ω_{R1}-ω_{R2} (R1 and R2 stand for ruby luminescence lines) and (b) the FWHM of the R1 line of the ruby piece used in the experiment as a pressure gauge.

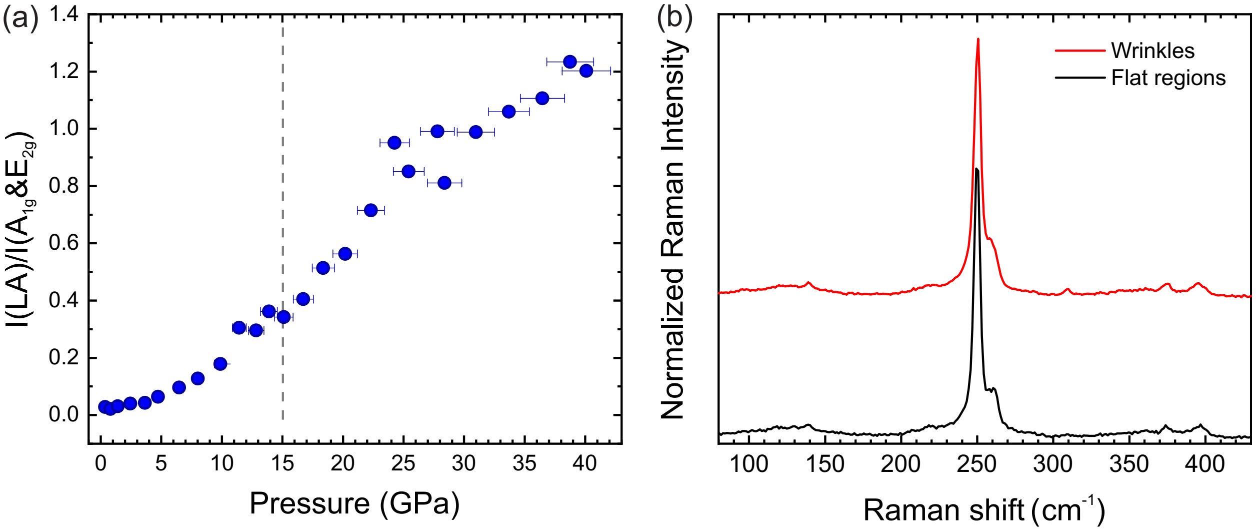

Figure S 4: (a) Pressure dependence of the LA/A_{1g}&E_{2g} intensity ratio. (b) Raman spectra obtained by summing multiple pixels from wrinkle (red trace) and flat (black trace) regions.
